# Intergenerational support and subjective wellbeing among oldest-old in China: the moderating role of economic status

**DOI:** 10.1186/s12877-021-02204-y

**Published:** 2021-04-15

**Authors:** Fanghong Huang, Peipei Fu

**Affiliations:** 1 School of Economics, Shandong University, Jinan, 250100 China; 2 Centre for Health Management and Policy Research, School of Public Health, Cheeloo College of Medicine, Shandong University, Jinan, 250012 China; 3 NHC Key Lab of Health Economics and Policy Research (Shandong University), Jinan, 250012 China

**Keywords:** Intergenerational support, Oldest-old, Subjective wellbeing, Moderating effect, Economic status

## Abstract

**Backgrounds:**

The oldest-old population is increasing sharply in China, and intergenerational support has been their primary source of caregiving. Although intergenerational support has been found to be associated with wellbeing of older people in previous study, most analysis were from the perspective of children’s characteristics and exchange patterns. This study aims to investigate the impact of different types of intergenerational support on subjective wellbeing among Chinese oldest-old and the variation across groups of different economic status, based on their five-tier of needs (physiological needs, safety needs, love/belonging needs, esteem needs, and self-actualization needs).

**Methods:**

We included older adults aged ≥ 80 years from the 2018 Chinese longitudinal Healthy Longevity Survey (CLHLS). We assessed older people’s subjective wellbeing by their life satisfaction and psychological health. We evaluated four types of intergenerational support: parents provide financial support, receive financial, instrumental and emotional support. We applied binary logistic regression analysis to analyze the association between different intergenerational support and older people’s subjective wellbeing and the moderating effect of self-rated economic status on this relationship.

**Results:**

A total of 8.794 participants were included, with a mean age of 91,46 years (standard deviation:7.60). Older adults who provide financial support (OR: 1.37, 95% CI: 1.01, 1.85) and receive emotional support (OR: 1.99, 95% CI: 1.40, 2.83) report better subjective wellbeing. However, receiving instrumental support depressed psychological health (OR: 0.67, 95% CI: 0.56, 0.79) while improved life satisfaction (OR: 1.42, 95% CI: 1.04, 1.55). Receiving emotional support promoted parents’ psychological health among all combinations of support, and receiving all the three types together raised their subjective wellbeing most.

**Conclusions:**

Our study recognizes that higher level of subjective wellbeing for oldest-old is related to providing financial support, receiving emotional and certain instrumental support. In addition, higher economic status can moderate these associations.

**Supplementary Information:**

The online version contains supplementary material available at 10.1186/s12877-021-02204-y.

## Background

Population aging is a ubiquitous problem worldwide, especially in China. Rapid declines in mortality and fertility are expected to accelerate the aging process. The population aged 65 and over reached 118.94 million in 2010 and 172.26 million in 2020, accounting for 8.9 and 12.0% of the total population in China respectively [1, 2]. Most importantly, people aged 80+ (the oldest-old) were estimated to be 26.6 million and accounted for 1.8% in 2020, but they are projected to increase considerably and reach 111.5 million in 2050 according to a forecast from the United Nations (UN). The proportion will climb from 1.8% in 2020 to 8.3% in 2050 [2].

Ageing is usually associated with declining economic resources, decreasing cognitive ability, deteriorating physical health and weakening social support [3]. Progressively increasing with age, frailty and multimorbidity have been suggested as risk factors for psychological health [4]. These changes in life circumstances suggest that aging might be related with declining well-being among the older adults. With increasing life expectancy, maintaining both longevity and a high level of wellbeing is regarded as an important indicator of successful aging. Consequently, the wellbeing of oldest-old group should be attached more importance. The measurement and influencing factors of subjective wellbeing have been studied in previous researches. In China, Xing firstly [5] systematically expounded the measurement of subjective well-being and summarized it into two indicators: one is in the sense of life satisfaction, which defines subjective well-being as people’s cognitive evaluation of their own life satisfaction; another is in the sense of psychological health, which points out that subjective well-being depends on the balance of positive and negative emotions in a certain period of time. Evidence from China suggested that among oldest-old, factors correlated with life satisfaction include sex, education, place of residence, self-rated health status, regular physical examination, perceived relative economic status, access to social security provisions, commercialized insurances, living arrangements, and number of social services available in the community, while the traditional role of family in supporting the older persons continued to be an important contributor [6]. As one ages, the social network tends to shrink [7], physical function deteriorates uncontrollably especially for oldest-old, family support continues to be a primary source of care and support for old people in many developing countries [8], motivating our attention to the relationship between intergenerational support and subjective well-being among oldest-old.

The intergenerational support is commonly distinguished in three types: financial, instrumental and emotional support [9, 10]. Prior studies showed that material support and spiritual support within family can replace each other in promoting older individual’s life satisfaction [11], while some pointed out that non-financial support from offspring may be more important factors in promoting Chinese oldest-old’s subjective wellbeing [12]. Furthermore, support provision and receipt can have different influences on older wellbeing. Providing financial support to children may give older parents higher levels of self-esteem and independence, which is beneficial to their wellbeing [13]. While receiving support from adult children appeared to lead to better health and wellbeing of older parents [14], it could endanger feelings of dependence and loss of autonomy [15].

In spite of abundant studies about the relationship between intergenerational support and wellbeing of older people, surprisingly few of them consider variation across older adults of different economic status. Economic resource is the basic guarantee of residents’ life, and directly affect the quality of life and older individuals’ wellbeing. Guo found that income played as a moderator in the association between psychological well-being of older adults and chronic diseases [16]. However, previous research pointed out that income has a positive impact on wellbeing at a relatively lower level, because material consumption makes great difference in improving people’s life; yet with income increasing, non-material factors become more important in individual’s life relative to economic resources. The findings are corresponding with Maslow’s hierarchy of needs theory [17, 18], which comprises a five-tier model of human needs (physiological needs, safety needs, love/belonging needs, esteem needs, and self-actualization needs). Lower-order needs relying on economic resources have a higher prepotency than higher-order needs, while higher-order satisfaction is pursued once lower-order satisfaction is achieved. Therefore, we hypothesize that the association among different types of support, which aims at meeting different order of needs, and older individuals’ subjective wellbeing varies across different economic-status groups.

In order to fill these research gaps, this study applies logistic regression analysis to investigate the relationship between intergenerational support and the two indicators of subjective wellbeing: life satisfaction and psychological health. With regards to types of support, we examined all types in the receiving flow and focused only financial support in the providing flow. Because while instrumental support is beyond capacity for oldest-old mostly in poor health, emotional support is provided and received simultaneously, whose flowing direction is hard to distinguish. We also combined the three types of support in the receiving flow to explore their interacting associations. Additionally, variation in the association between intergenerational support and subjective wellbeing would be scrutinized.

## Methods

### Data source

The data for this study was used from the 2018 Chinese longitudinal Healthy Longevity Survey (CLHLS), the ongoing national representative longitudinal survey established in 1998. Aiming to shed new light and better understanding of the determinants of healthy longevity of human beings, provide information evidence for scientific research as well as health and population-aging policy, the CLHLS has conducted seven waves, covering 22 of the 31 provinces in China and using a stratified multistage cluster sampling design [19]. It conducted a super-proportional sampling on the male and urban older groups. We did not apply sampling weights in the regression models because the CLHLS weight variable was unable to reflect the national population distributions with respect to variables other than age, sex, and urban/rural residence [20, 21].

A total of 15,874 older adults were interviewed (median age 85 years, females making up 56, and 22% of these were interviewed in earlier waves). This study focused on the 8794 oldest-old adults aged 80 or over (mean (M) = 91.46; standard deviation (SD) = 7.60). Missing values and data of answers like “don’t know”, “not applicable” were excluded out of the sample. Moreover, some samples with “unable to answer” may bring bias into our measures of subjective wellbeing, since the respondents cannot evaluate their life and personality because of impairment of cognitive function. Diseases related to impairment of cognitive function include dementia, Parkinson’s disease, stroke and cerebrovascular disease [22]. The Community Screening Instrument for Dementia (CSI-D cognition) in CLHLS questionnaire was used to determined dementia if none of the questions was answered correctly. Therefore, respondents of “unable to answer” with impairment of cognitive function were excluded out of our sample.

### Measures

Following extant researches [5, 6, 23], in combination with the design of the CLHLS questionnaire, a set of variables were chosen for data analysis (Table 1).

**Table 1:** Description of study variables

Variables	Description
Life satisfaction	0 = Bad, 1 = Good
Psychological health	0 = Depressive, 1 = Normal
Provide financial support	0 = No, 1 = Yes
Receive financial support	0 = No, 1 = Yes
Receive instrumental support	0 = No, 1 = Yes
Receive emotional support	0 = No, 1 = Yes
Combination of receiving support	1 = No support, 2 = Financial support, 3 = Instrumental support, 4 = Emotional support, 5 = Financial and instrumental support, 6 = Financial and emotional support, 7 = Instrumental and emotional support, 8 = Financial, instrumental and emotional support
Age (years)	Validated age of older adult
Gender	0 = Male, 1 = Female
Current residence	The current residential area of the interviewed elderly (0 = Rural, 1 = Urban)
Current marital status	1 = Currently married and living with spouse, 2 = Never married, separated and divorced, 3 = Widowed
Income	Total income of the older adult’s household last year
Self-rated economic status	How does the older rate his economic status compared with other local people? (1 = Bad, 2 = Average, 3 = Good)
Years of schooling	Years of schooling
Number of living children	Number of living children
Co-residence	Whether living with adult children (0 = No, 1 = Yes)
ADL	For the last 6 months, whether limited in activities because of health problem (0 = Not limited, 1 = Limited)
Chronic disease	Whether suffering chronic disease (0 = No, 1 = Yes)
Social security insurance	Whether having any social security and social insurance now (0 = No, 1 = Yes)
Medical insurance	Whether having any of urban employee/urban resident/new rural cooperative/commercial medical insurance now (0 = No, 1 = Yes)
Old-age insurance	Whether having any of public/private/commercial old-age insurance now (0 = No, 1 = Yes)
Community service	Whether having any community service now (0 = No, 1 = Yes)

#### Dependent variables

In this study, we used life satisfaction and psychological health as important indicators to reflect the subjective wellbeing of older adults. Life satisfaction was assessed by asking respondents to rate their lives at present on a five-point scale -- very good (1) to very bad (5). We recoded the items and defined respondents who chose “, bad, very bad” regarding their life satisfaction as bad (0), who chose “very good, good, average” regarding their life satisfaction as good (1). Psychological health of the older adults is measured by the depression symptom. The 10-item Center for Epidemiologic Studies Short Depression Scale (CES-D) was adopted to measure the depression symptom, scoring from 0 to 30 with a cutoff point of 10, to distinguish depressive (0) and normal groups (1) [23].

#### Key explanatory variables

There were four key explanatory variables in this study: provide financial support, receive financial, instrumental and emotional support (as three types of intergenerational support flowing in opposite directions). Providing financial support was evaluated by stating the amount of money (including cash and value of materials) that the elder gave last year to his children and their spouses both living and not living with him; while receiving financial support was estimated by such asset older adults received in turn. Receiving instrumental support was assessed by primary caregiver when the elder need assistance in daily activities and the number of hours that adult children help him last week. Receiving emotional support was evaluated by the person to whom older adults usually talk frequently in daily life and whom they talk first when they need to share their thoughts. Choices on adult children, grandchildren and their spouses would be regarded as receiving the support. All four variables were divided into “Yes” and “No” to show whether the intergenerational support was provided to the recipient. Furthermore, in order to explore the interacting associations among receiving three types of support, we generated a new variable as combination of the three types of support: receiving none of the supports (1); receiving only one type of support among “financial (2), instrumental (3), emotional (4)”; receiving two types of the three categories: “financial and instrumental (5), financial and emotional (6), instrumental and emotional (7)”; and receiving all the three types (8).

#### Confounding variables

The potential confounding variables consisted of demographic, physical condition, and other covariates [6]. Demographic variables included age, gender (male and female), current residence (urban and rural), current marital status (currently married and living with spouse; widowed; never married, separated and divorced), self-rated economic status (good, average, bad), income, years of schooling, number of living children and co-residence (whether or not living with children). Additionally, whether the older individual has a chronic disease (Yes and No) and his Activities of Daily Living (ADL) is limited (Limited and Not limited) were taken as physical condition variables. Apart from intergenerational support within the family, other covariates like social security insurance, medical insurance, old-age insurance and community service, either provided by the government or private corporation, would offer older adults a sense of security in the aging life so as to increase their wellbeing.

### Statistical analysis

**Fig. 1:**
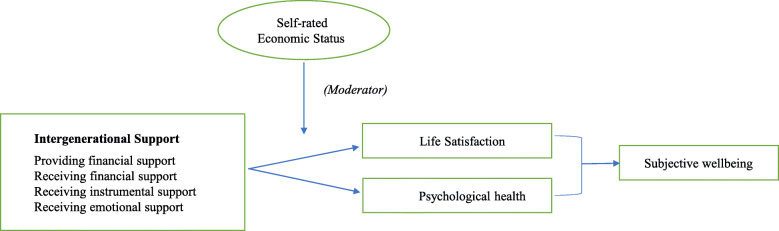
The association between intergenerational support and subjective wellbeing moderated by self-rated economic status

Descriptive analysis was carried out for intergenerational support, demographic data, physical condition, and other covariates. Binary logistic regression analysis (Fig. 1) was used to analyze the influence of intergenerational support on life satisfaction and psychological health of older adults (Tables 3 and 4). First, crude odds ratios (ORs) and 95% confidence intervals (CIs) for key explanatory variables (provide financial support, receive financial, instrumental and emotional support) were calculated in model 1 and 3. Second, confounding variables including demographic data, physical condition and other covariates were controlled in model 2 and 4. To capture how the three types of support interact and compliment to each other in the receiving flow, we then introduced the combination variable and performed the logistic regression in model 5 and 6. We also tried to investigate whether the associations of intergenerational support types vary across different economic-status older groups, we then introduced interaction terms between intergenerational support and self-rated economic status in model 7 and 8 (Supplementary Table 1). Robust standard errors were implemented to adjust for heteroscedasticity. Test of multicollinearity for all variables resulted in the variance inflation factor (VIF) scores ranging from 1.04 to 8.74, indicating no concerns about multicollinearity. Furthermore, in order to test the robustness of the results, we conducted several sensitive analyses: as the excluding proportion for “unable to answer” in CLHLS is relatively high and the respondents are mostly in poorer health, we applied multiple imputation for missing values to perform the estimation (Supplementary Table 2, 3). Regarding the variable of life satisfaction, while the original ordinal answers were coded into dichotomous ones, multinominal logistic regression was applied to analyze the original category (Supplementary Table 4, 5 and 6). The sensitivity analysis shows a consistent finding with the main analyses. Finally, all statistics procedures were performed by using Stata MP 14.0 (StataCorp, College Station, TX, USA).

## Results

Table 2 presents the distribution characteristics of the research variables. Generally, respondents rated their life satisfaction into 2 categories: good (96.71%), bad (3.29%), while their psychological health was classified to be normal (87.78%) and depressive (12.22%). For the key explanatory variables, only 35.35% of the respondents reported to provide financial support to their children, while a mass of 76.44% receive those in turn. For both instrumental and emotional support from adult children, the achieving choice composes mass of the sample (67.81 and 89.32%). The mean age of study sample was 91.46, females represented more as 57.89%. More respondents (55.77%) currently resided in urban areas, 72.94% were widowed, 73.45% were not living with their children. The average total income of their household last year was 41,476.03. They had averagely 2.36 years of schooling and the mean number of living children was 3.77. 82.13% of the participants suffered from chronic diseases, however, 58.68% of the sample were not limited for ADL. Other covariates differed greatly among the sample: social security insurance and medical insurance covered a wide range of the sample, by 90.88 and 85.29% separately; community service was provided relatively less to the participants as 64.40%; Majority of the respondents did not have old-age insurance (74.01%).

**Table 2:** Distribution of study variables

Variables	Mean ± SD or n (%)
Total	8794
Life satisfaction	
Good	8315 (96.71)
Bad	283 (3.29)
Psychological health	
Normal	6467 (87.78)
Depressive	900 (12.22)
Provide financial support	
Yes	2727 (35.35)
No	4987 (64.65)
Receive financial support	
Yes	6166 (76.44)
No	1900 (23.56)
Receive instrumental support	
Yes	4750 (67.81)
No	2255 (32.19)
Receive emotional support	
Yes	7786 (89.32)
No	931 (10.68)
Combination of receiving support	
No support	303 (3.45)
Financial support	351 (3.99)
Instrumental support	150 (1.71)
Emotional support	883 (10.04)
Financial and instrumental support	204 (2.32)
Financial and emotional support	2507 (28.51)
Instrumental and emotional support	1292 (14.69)
Financial, instrumental and emotional support	3104 (35.30)
Age (years)	91.46 ± 7.60
Gender	
Male	3703 (42.11)
Female	5091 (57.89)
Current residence	
Urban	4904 (55.77)
Rural	3890 (44.23)
Current marital status	
Currently married and living with spouse	2162 (24.83)
Widowed	6350 (72.94)
Never married, separated and divorced	194 (2.23)
Income	41,476.03 ± 36,827.95
Years of schooling	2.36 ± 3.85
Number of living children	3.77 ± 1.88
Co-residence	
Yes	2312 (26.55)
No	6395 (73.45)
ADL (Activities of daily living)	
Limited	3634 (41.32)
Not limited	5160 (58.68)
Chronic disease	
Yes	6035 (82.13)
No	1313 (17.87)
Social security insurance	
Yes	6507 (90.88)
No	653 (9.12)
Medical insurance	
Yes	7185 (85.29)
No	1239 (14.71)
Old-age insurance	
Yes	2286 (25.99)
No	6508 (74.01)
Community service	
Yes	5425 (64.40)
No	2999 (35.60)

The separate analysis of types of intergenerational support on life satisfaction and psychological health (model 1, 3) are presented in Table 3, followed by introduction of other covariates (model 2, 4). Older adults who provided financial support to children, [odds ratio (OR): 1.369, 95% confidence interval (CI): 1.011, 1.853], received instrumental and emotional support back (OR: 1.419, 95% CI: 1.036, 1.943; OR: 1.985, 95% CI: 1.395, 2.826) are more likely to report better life satisfaction. Model 3 and 4 specifies the positive association of providing financial support (OR: 1.192, 95% CI: 0.995, 1.429) and receiving emotional support (OR: 1.457, 95% CI: 1.165, 1.823) on respondents’ psychological health, and receiving instrumental support suggests a negative relationship (OR: 0.665, 95% CI: 0.561, 0.789) with psychological health in model 3.

**Table 3:** Logistic regression on Chinese oldest-old’s Life satisfaction and Psychological health

VARIABLES	Life satisfaction (OR, 95% CI)		Psychological health, (OR, 95% CI)	
	Model 1	Model 2	Model 3	Model 4
Intergenerational support variables				
Provide financial support	1.715*** (1.279–2.300)	1.369** (1.011–1.853)	1.364*** (1.154–1.612)	1.192** (0.995–1.429)
Receive financial support	0.885 (0.663–1.181)	0.889 (0.652–1.212)	0.911 (0.753–1.101)	0.928 (0.760–1.134)
Receive instrumental support	1.086 (0.823–1.432)	1.419** (1.036–1.943)	0.665*** (0.561–0.789)	0.874 (0.722–1.056)
Receive emotional support	2.345*** (1.730–3.178)	1.985*** (1.395–2.826)	1.518*** (1.229–1.873)	1.457*** (1.165–1.823)
Age		0.997 (0.979–1.016)		1.010* (0.999–1.022)
Gender		0.840 (0.633–1.114)		0.868 (0.733–1.028)
Current residence		1.098 (0.862–1.398)		0.995 (0.855–1.158)
Current marital status				
Never married, separated and divorced		0.647 (0.338–1.239)		0.638** (0.410–0.990)
Widowed		0.860 (0.608–1.215)		0.689*** (0.561–0.846)
Income		1.000*** (1.000–1.000)		1.000*** (1.000–1.000)
Year of schooling		1.032 (0.985–1.082)		1.024* (0.999–1.050)
Number of living children		1.102*** (1.034–1.175)		1.063*** (1.021–1.106)
Co-residence		1.144 (0.845–1.548)		1.149 (0.969–1.363)
ADL		0.440*** (0.341–0.569)		0.408*** (0.350–0.476)
Chronic disease		0.820 (0.574–1.172)		0.797* (0.633–1.004)
Social security insurance		1.286 (0.835–1.980)		1.104 (0.843–1.445)
Medical insurance		0.865 (0.602–1.245)		0.860 (0.694–1.066)
Old-age insurance		1.044 (0.791–1.379)		1.153 (0.971–1.369)
Community service		1.193 (0.921–1.545)		1.020 (0.873–1.192)

*** *p* < 0.01, ** *p* < 0.05, * *p* < 0.1

Table 4 presents the results of combinations of types of intergenerational support on life satisfaction and psychological health (model 5, 6). Older individuals who received only financial support reported a poorer life satisfaction (OR: 0.513, 95% CI: 0.267, 0.985), and those who received both of the instrumental and emotional support responded a better one (OR: 1.730, 95% CI: 0.924, 3.241). Those who received all three types had a rather positive association with their life satisfaction (OR: 2.045, 95% CI: 1.139, 3.670). In model 6, those who received only emotional support presented a positive relationship on older adults’ psychological health, while added the variable of financial support reduced the association. Additionally, introducing the variable of the instrumental support could improve the positive association, indicating that received all three supports brought a better psychological health.

**Table 4:** Logistic regression of combination on Chinese oldest-old’s Life satisfaction and Psychological health

VARIABLES	Life satisfaction (OR, 95% CI)	Psychological health, (OR, 95% CI)
	Model 5	Model 6
Combination of receiving support variables (No support = 1)		
Financial support	0.513** (0.267–0.985)	1.134 (0.700–1.835)
Instrumental support	0.662 (0.311–1.410)	1.011 (0.583–1.754)
Emotional support	1.378 (0.706–2.681)	1.689** (1.112–2.565)
Financial and instrumental support	1.741 (0.658–4.610)	1.536 (0.855–2.760)
Financial and emotional support	1.461 (0.816–2.617)	1.565** (1.076–2.277)
Instrumental and emotional support	1.730* (0.924–3.241)	1.343 (0.904–1.994)
Financial, instrumental and emotional support	2.045** (1.139–3.670)	1.583** (1.085–2.309)
Age	0.997 (0.979–1.015)	1.010 (0.998–1.021)
Gender	0.841 (0.629–1.123)	0.867* (0.732–1.026)
Current residence	1.119 (0.871–1.438)	1.009 (0.866–1.175)
Current marital status		
Never married, separated and divorced	0.594 (0.308–1.145)	0.659* (0.419–1.035)
Widowed	0.854 (0.607–1.202)	0.686*** (0.559–0.840)
Income	1.000*** (1.000–1.000)	1.000*** (1.000–1.000)
Year of schooling	1.035 (0.987–1.086)	1.027** (1.002–1.052)
Number of living children	1.094*** (1.024–1.168)	1.054*** (1.013–1.097)
Co-residence	1.169 (0.864–1.581)	1.153 (0.972–1.368)
ADL	0.436*** (0.335–0.565)	0.401*** (0.343–0.468)
Chronic disease	0.846 (0.590–1.213)	0.805* (0.638–1.015)
Social security insurance	1.258 (0.813–1.946)	1.095 (0.837–1.433)
Medical insurance	0.856 (0.592–1.238)	0.853 (0.688–1.057)
Old-age insurance	1.032 (0.782–1.363)	1.150 (0.969–1.366)
Community service	1.191 (0.919–1.544)	1.011 (0.865–1.181)

*** *p* < 0.01, ** *p* < 0.05, * *p* < 0.1

## Discussion

The current study investigates relationships between intergenerational support and subjective wellbeing (life satisfaction and psychological health) among older adults. Based on the cross-sectional 2018 CLHLS data, we confirmed that different types of intergenerational support show different patterns of impact on life satisfaction and psychological health of older adults in China. In addition, we provided new evidence that the three types of support interact on each other in the receiving flow, and these relationships were moderated by self-rated economic status after adjusting for demographic factors, physical conditions and other covariates.

Regarding financial support, our results found that the positive association between providing financial transfer to children and Chinese older adults’ life satisfaction. Our study is consistent with results of a study in Hong Kong that those under-benefited (support provided exceeds what he/she received) reported the highest level of life satisfaction [24]. Furthermore, a study among Chinese older adults demonstrates that engaging in contributory behaviors is positively associated with life satisfaction. “Contributory behaviors” acknowledges older adults’ abilities to contribute to their family, community and society through meaningful activities, involving providing economic, labor and emotional support to their adult children [25]. In the current study on oldest-old, a majority of interviewees suffers from health problems (e.g., chronic disease: 82.16%) and nearly half of them are limited with ADL, therefore financial support is regarded as their representative “contributory behavior”. They feel valuable through the show of their wisdom and ability, and the involvement of fulfilling social responsibilities and obligations would meet their self-actualization need, which stays at the highest order of Maslow’s hierarchy. In contrast, receiving financial support have no significant relationship with neither life satisfaction nor psychological health. The improvement of economic status from children’s financial support brings limited positive effect on wellbeing, when most Chinese people have their lower-order needs of food, health and safety satisfied. The contributory behavior of providing financial support motivated by family love and commitment helps break the bottleneck on raising wellbeing, as older people always express great pleasure and satisfaction in possessing the capacity. In addition, receiving only financial support may lead to a worse life satisfaction when we tried to explore the influence of combination. Current result coincides with a Turkish study that life satisfaction among older adults is negatively associated with the amount of help they received from adult children [26]. Although Chinese older adults usually rely on children’s financial support after retirement [27] and meet their safety need consequently, they were concerned about being a burden to their children [28]. In other words, older parents are reluctant to receive excessive financial support from children at the price of unduly troubling their children, which reflects the altruism and love of human nature in parents.

In current study, older adults’ life satisfaction is significantly improved by receiving emotional support. It echoes the discovery that emotional support, both received and provided, is positively related to self-rated health of older people [29]. Rating better economic status plays as a positive moderator in the association of receiving emotional support with life satisfaction. Older adults are motivated to pursue higher-order needs in terms of love/belonging and esteem after satisfying lower ones. Existing findings have shown that receiving emotional support helps older people better adjust to stressful events [30] and promote their self-identification against damage on esteem from health problems. Although the positive impact on their psychological health is not significant, older people’ life satisfaction achieves a remarkably improvement.

The study observes the primary importance of receiving instrumental support in enhancing older adult’s life satisfaction but depressing psychological health. Other research found that receiving instrumental support has either no or negative relation with older wellbeing [31, 32]. In fact, instrumental support involves both lower and higher order needs. With regards to lower needs of physiological and safety, oldest-old turn to the closest source of support in caring for acute and chronic diseases and seeking medical assistance. Social welfare and medical service in China can hardly meet the inordinate demands, especially in rural areas. Indeed, virtually all frail older adults in China—both those in rural and urban areas—rely on their children or other relatives for instrumental assistance and personal care [33]. Therefore, receiving instrumental support within family can greatly improve life satisfaction. Oppositely, the negative association of wellbeing is consistent with the depressive symptom of psychological health. For the study sample of oldest-old, which implies a majority of poor health, instrumental support is likely to be viewed as a signal of autonomy or dependency in their later life. A typical question in the Depression (CES-D) Scale in CLHLS is presented as “Do you feel the older you get, the more useless you are, and have trouble doing anything”. When the depression comes to be the obstacles of achieving esteem and self-actualization, older adults suffer from decreasing wellbeing against raising life satisfaction. The moderating role of economic status is revealed that receiving instrumental support reduce both life satisfaction and psychological health among “good” and “average” group. Because adequate economic resources limit the room for improvement in life satisfaction by providing complete medical and care services. Excessive instrumental support only works on exacerbating the depression symptom.

With regard to the combination of three types of support, our results manifest that receiving only financial support is negatively related to life satisfaction, while receiving other two types reverse the relationship to positive one. Study on Chinese older people’s perception of filial piety reveals that they expect least on financial support from children while the significance of receiving emotional support outweighs that of material support [34]. According to the contextual Turkish example, older women do not wish to burden their children but enjoy being thought of and valued [26]. Nevertheless, older adults who receive all the three types of support are expected to be more satisfied with their life, since a complete range of support play a better role in promoting life satisfaction. Receiving emotional support matters most in improving older parents’ psychological health, despite the offsetting effect after introducing the financial support to parents, as an impairment to their self-esteem. With the adding of instrumental support, full types of support help improve older parents’ psychological health together in a similar way.

In fact, as the development of Chinese economy in recent years, people’s wellbeing improves remarkably with richer economic condition. Therefore, on one hand, the negative association between receiving financial support and older adults’ life satisfaction indicates parents’ diminishing marginal utility of financial resources, as their pensions, social security insurance (90.87%) and medical insurance (85.29%) support most of life expenses. Meanwhile, their intense will to provide financial support for children and demand for emotional communications reflect their persistence in seeking satisfaction in higher-order needs in terms of spiritual aspects: sense of belonging, esteem and self-actualization, to gain extra wellbeing. On the other hand, for oldest-old, instrumental support significantly improves their life satisfaction in satisfying lower needs of physical and safety as aged, while impairing their psychological health in depressing their esteem and self-actualization needs.

At present, older adults’ high need of different types of intergenerational support and irreplaceable status of family in Chinese’s life determine that home-based care service, rather than institution like nursing home, should be the mainstream of aging provision. However, it is discovered that family support shows a significantly negative impact on adult children’s working opportunities and time [35]. Many adult children, especially for the only child in family, find it not cost-effective to give up work for caring parents and turn to social care service instead. One of our confounding factors, community service also promotes older adults’ life satisfaction in the same way as family support. Over the last two decades, the proportion of the population over 65 years, which suggests a higher need of social caregiving service for older people [36]. Although the demand of social caregiving service booms and function of family support weakens, high-quality care service project is in sharply short supply, as elderly care service industry and the long-term care system are still in their infancy and the government has not well prepared for coping with this challenge [36]. Chinese society is facing a strict strait on the requirement of social division on caregiving services for older adults, but the measures are not adequate at all [37].

There are some policy implications for both the family and community side. For one thing, government should invest more resources to strengthen older adults’ social security and medical insurance, providing robust financial support to help reduce family’s burden. Meanwhile, social policy should encourage frequent family visiting and communication to maintain adequate emotional support. For another thing, both public and commercial older care service industry should be potently promoted. Available type of public service including personal daily care, home visits, psychological consulting, daily shopping, social and recreation activities, legal aid, health education and neighboring relations, would cover nearly every aspect of older adults’ life and meet different types of needs. As demand for specific care service increase, policy should also encourage the development of home-based care service market. A mature market with plenty of competitive suppliers must promote emergence of high-quality and low-price service products and facilitate successful aging.

The present study is no free of limitations. First, although our research used the latest cross-sectional data in 2018 CLHLS on the purpose of finding out the up-to-date result. Due to the cross-sectional nature of data, no causal inferences can be drawn from our study. Second, our measure of intergenerational support was a rather simplistic binary variable indicating ‘yes’ or ‘no’. We do not consider the amount or level of that support. Previous study has found that moderate amounts of intergenerational support are beneficial to older parents while excessive support may be harmful [15]. Third, we were unable to take control of the characteristics of the adult children such as their needs, geographic proximity, economic status and possession of younger children (grandchildren for older adults). For example, studies found that financial transfers from parents were directed to young, unmarried family members, particularly females and those with less income [38] or to adult children with younger children [39]. Other adult children’s characteristics could be taken into consideration in the later studies. Finally, although we have controlled as possible as the covariates, the association between receiving support and subjective wellbeing still have the endogeneity issue possibly due to the nature of cross-sectional dataset, for example, older adults of poorer health need certain support like instrumental helps.

## Conclusions

Despite some limitations, the present study has provided strong evidence for impact of intergenerational support on oldest-old’s subjective wellbeing. The importance of providing more emotional support to all groups of older people, rather than simply financial transfer implicates that higher-order demands of oldest-old should be paid more attention by their children and government. With oldest-old population soaring, policies are suggested to raise family instrumental support by encouraging both public and commercial services, especially to those in poor economic status, which needs future deeper studies and wider trials.

## Supplementary Information

**Additional file 1: Supplementary Table 1**. Moderating role of self-rated economic status. **Supplementary Table 2**. Logistic regression on Chinese oldest-old’s Life satisfaction and Psychological health (After imputed for sample with “unable to answer”, *n* = 10,427). **Supplementary Table 3**. Logistic regression of combination on Chinese oldest-old’s Life satisfaction and Psychological health (After imputed for sample with “unable to answer”, n = 10,427). **Supplementary Table 4**. Multinominal logistic regression on Chinese oldest-old’s Life satisfaction with original category (Model 1). **Supplementary Table 5**. Multinominal logistic regression on Chinese oldest-old’s Life satisfaction with original category (Model 2). **Supplementary Table 6**. Multinominal logistic regression on Chinese oldest-old’s Life satisfaction with original category (Model 4).

## Data Availability

The datasets generated and/or analyzed during the current study are available in the Peking University Open research data repository, https://opendata.pku.edu.cn/dataset.xhtml?persistentId=doi:10.18170/DVN/WBO7LK

## Abbreviations

CLHLS: Chinese longitudinal Healthy Longevity Survey
ADL: Activities of Daily Living
