# Supplementary material for: Intergenerational support and subjective wellbeing among oldest-old in China: the moderating role of economic status
Source: BMC Geriatr. 2021 Apr 15;21:252. doi: 10.1186/s12877-021-02204-y (PMC8051050; doi:10.1186/s12877-021-02204-y)
Supplement: Supplementary file 1 — Additional file 1: Supplementary Table 1. Moderating role of self-rated economic status. Supplementary Table 2. Logistic regression on Chinese oldest-old’s Life satisfaction and Psychological health (After imputed for sample with “unable to answer”, n = 10,427). Supplementary Table 3. Logistic regression of combination on Chinese oldest-old’s Life satisfaction and Psychological health (After imputed for sample with “unable to answer”, n = 10,427). Supplementary Table 4. Multinominal logistic regression on Chinese oldest-old’s Life satisfaction with original category (Model 1). Supplementary Table 5. Multinominal logistic regression on Chinese oldest-old’s Life satisfaction with original category (Model 2). Supplementary Table 6. Multinominal logistic regression on Chinese oldest-old’s Life satisfaction with original category (Model 4). [file 12877_2021_2204_MOESM1_ESM.docx]

Supplementary Table 1. Moderating role of self-rated economic status

| VARIABLES | Life satisfaction (OR, 95% CI) | Psychological health, (OR, 95% CI) |
| --- | --- | --- |
|  | Model 7 | Model 8 |
| Intergenerational support variables | | |
| Provide financial support | 1.525 (0.894 – 2.600) | 1.068 (0.702 – 1.625) |
| Receive financial support | 0.947 (0.586 – 1.532) | 1.036 (0.677 – 1.580) |
| Receive instrumental support | 1.902*** (1.196 – 3.024) | 1.328 (0.893 – 1.974) |
| Receive emotional support | 1.235 (0.753 – 2.026) | 1.308 (0.858 – 1.993) |
| Age | 0.993 (0.974 – 1.012) | 1.009 (0.997 – 1.021) |
| Gender | 0.812 (0.600 – 1.101) | 0.853* (0.718 – 1.014) |
| Current residence | 1.113 (0.859 – 1.442) | 0.999 (0.857 – 1.165) |
| Current marital status |  |  |
| Never married, separated and divorced | 0.643 (0.327 – 1.264) | 0.669* (0.421 – 1.061) |
| Widowed | 0.844 (0.591 – 1.205) | 0.681*** (0.554 – 0.839) |
| Self-rated economic status (0 = Bad) |  |  |
| Average | 7.805*** (3.527 – 17.272) | 3.810*** (2.193 – 6.619) |
| Good | 9.307*** (2.027 – 42.741) | 17.493*** (5.949 – 51.437) |
| Year of schooling | 1.007 (0.960 – 1.057) | 1.008 (0.983 – 1.033) |
| Number of living children | 1.039 (0.972 – 1.110) | 1.034 (0.993 – 1.076) |
| Co-residence | 1.283 (0.944 – 1.743) | 1.219** (1.025 – 1.450) |
| ADL | 0.505*** (0.384 – 0.664) | 0.435*** (0.371 – 0.509) |
| Chronic disease | 0.862 (0.594 – 1.252) | 0.809* (0.641 –1.021) |
| Social security insurance | 1.328 (0.851 – 2.071) | 1.093 (0.830 –1.440) |
| Medical insurance | 0.852 (0.584 – 1.243) | 0.869 (0.696 – 1.084) |
| Old-age insurance | 1.039 (0.781 – 1.383) | 1.146 (0.962 – 1.366) |
| Community service | 1.147 (0.877 – 1.498) | 1.008 (0.860– 1.183) |
| Interactions (Intergenerational support×Self-rated economic status) | | |
| Provide financial support$\times$Average | 0.730 (0.370 – 1.439) | 1.110 (0.698 – 1.766) |
| Provide financial support$\times$Good | 0.981 (0.261 – 3.695) | 0.940 (0.513 – 1.724) |
| Receive financial support$\times$Average | 0.935 (0.479 – 1.826) | 0.924 (0.583 – 1.466) |
| Receive financial support$\times$Good | 0.891 (0.224 – 3.540) | 0.809 (0.411 – 1.596) |
| Receive instrumental support$\times$Average | 0.599* (0.324 – 1.107) | 0.585** (0.374 – 0.912) |
| Receive instrumental support$\times$Good | 0.441 (0.123 – 1.579) | 0.484** (0.243 – 0.963) |
| Receive emotional support$\times$Average | 1.525 (0.752 – 3.092) | 1.054 (0.641 – 1.732) |
| Receive emotional support$\times$Good | 3.812** (1.078 – 13.479) | 0.637 (0.240 – 1.696) |

*** p<0.01, ** p<0.05, * p<0.1

Supplementary Table 2. Logistic regression on Chinese oldest-old’s Life satisfaction and Psychological health

(After imputed for sample with “unable to answer”, n = 10,427)

| VARIABLES | Life satisfaction (OR, 95% CI) | | Psychological health, (OR, 95% CI) | |
| --- | --- | --- | --- | --- |
|  | Model 1 | Model 2 | Model 3 | Model 4 |
| Intergenerational support variables | | | | |
| Provide financial support | 1.926*** (1.430 – 2.595) | 1.435** (1.060 – 1.943) | 1.409*** (1.200 – 1.655) | 1.166* (0.978 – 1.389) |
| Receive financial support | 1.004 (0.781 – 1.292) | 1.008 (0.767 – 1.324) | 0.958 (0.796 – 1.153) | 0.980 (0.799 – 1.202) |
| Receive instrumental support | 0.879 (0.673 – 1.148) | 1.225* (0.920 – 1.631) | 0.607*** (0.512 – 0.721) | 0.813** (0.679 – 0.974) |
| Receive emotional support | 2.493*** (1.844 –3.370) | 2.128*** (1.492 – 3.035) | 1.435*** (1.125 – 1.829) | 1.317*** (1.009 – 1.717) |
| Age |  | 0.992 (0.974 – 1.011) |  | 1.007 (0.996 – 1.019) |
| Gender |  | 0.887 (0.670 – 1.175) |  | 0.848* (0.713 – 1.008) |
| Current residence |  | 1.162 (0.906 – 1.492) |  | 1.001 (0.843 – 1.187) |
| Current marital status |  |  |  |  |
| Never married, separated and divorced |  | 0.682 (0.374 – 1.243) |  | 0.657* (0.431 – 1.006) |
| Widowed |  | 0.891 (0.638 – 1.244) |  | 0.696*** (0.561 – 0.852) |
| Income |  | 1.000*** (1.000 –1.000) |  | 1.000*** (1.000 – 1.000) |
| Year of schooling |  | 1.042* (0.996 – 1.091) |  | 1.004 (0.979 – 1.031) |
| Number of living children |  | 1.081** (1.016 – 1.150) |  | 1.064*** (1.018 – 1.113) |
| Co-residence |  | 1.116 (0.842 – 1.479) |  | 1.202* (0.997 – 1.448) |
| ADL |  | 0.394*** (0.309 – 0.502) |  | 0.398*** (0.341 – 0.476) |
| Chronic disease |  | 0.897 (0.649 – 1.239) |  | 0.758** (0.594 – 0.967) |
| Social security insurance |  | 1.191 (0.788 – 1.801) |  | 1.104 (0.827 – 1.474) |
| Medical insurance |  | 0.857 (0.595 – 1.236) |  | 0.824 (0.648 – 1.047) |
| Old-age insurance |  | 1.013 (0.798 – 1.285) |  | 1.162* (0.991 – 1.362) |
| Community service |  | 1.167 (0.907 – 1.502) |  | 1.016 (0.855 – 1.207) |

*** p<0.01, ** p<0.05, * p<0.1

Supplementary Table 3. Logistic regression of combination on Chinese oldest-old’s Life satisfaction and Psychological health

(After imputed for sample with “unable to answer”, n = 10,427)

| VARIABLES | Life satisfaction (OR, 95% CI) | Psychological health, (OR, 95% CI) |
| --- | --- | --- |
|  | Model 5 | Model 6 |
| Combination of receiving support variables (No support = 1) | | |
| Financial support | 0.552* (0.285 – 1.069) | 0.979 (0.599 – 1.600) |
| Instrumental support | 0.759 (0.399 – 1.447) | 0.868 (0.542 – 1.389) |
| Emotional support | 1.490 (0.767 – 2.893) | 1.305 (0.836 – 2.038) |
| Financial and instrumental support | 1.259 (0.584 – 2.716) | 1.122 (0.640 – 1.969) |
| Financial and emotional support | 1.704* (0.960 – 3.026) | 1.330 (0.891 –1.985) |
| Instrumental and emotional support | 1.597* (0.896 – 2.846) | 1.058 (0.704 – 1.590) |
| Financial, instrumental and emotional support | 1.938** (1.112 – 3.380) | 1.183 (0.796 – 1.758) |
| Age | 0.994 (0.976 – 1.012) | 1.007 (0.995 – 1.019) |
| Gender | 0.891 (0.672 – 1.180) | 0.851* (0.715 – 1.012) |
| Current residence | 1.179 (0.911 – 1.526) | 1.015 (0.856 – 1.203) |
| Current marital status |  |  |
| Never married, separated and divorced | 0.617 (0.335 – 1.135) | 0.658* (0.429 – 1.011) |
| Widowed | 0.888 (0.640 – 1.234) | 0.698*** (0.569 – 0.855) |
| Income | 1.000*** (1.000 – 1.000) | 1.000*** (1.000 – 1.000) |
| Year of schooling | 1.044* (0.997 – 1.094) | 1.006 (0.981 – 1.033) |
| Number of living children | 1.075** (1.011 – 1.144) | 1.058** (1.011 – 1.108) |
| Co-residence | 1.154 (0.874 – 1.523) | 1.208** (1.003 – 1.456) |
| ADL | 0.396*** (0.309 – 0.506) | 0.393*** (0.336 – 0.458) |
| Chronic disease | 0.922 (0.665 – 1.277) | 0.765** (0.599 – 0.975) |
| Social security insurance | 1.170 (0.775 – 1.766) | 1.094 (0.819 – 1.461) |
| Medical insurance | 0.852 (0.590 – 1.229) | 0.820 (0.645 – 1.041) |
| Old-age insurance | 1.031 (0.811 – 1.311) | 1.178 (1.005 – 1.381) |
| Community service | 1.162 (0.904 – 1.495) | 1.007 (0.848 – 1.195) |

*** p<0.01, ** p<0.05, * p<0.1

Supplementary Table 4. Multinominal logistic regression on Chinese oldest-old’s Life satisfaction with original category (Model 1)

| Variables | Model 1 (OR, 95% CI, Base category = Bad) | | | |
| --- | --- | --- | --- | --- |
|  | Very bad | Average | Good | Very Good |
| Intergenerational support variables | | | | |
| Provide financial support | 0.600 (0.200 – 1.798) | 1.307 (0.949 – 1.801) | 1.587*** (1.158 – 2.176) | 2.273*** (1.653 – 3.126) |
| Receive financial support | 0.904 (0.371 – 2.202) | 1.007 (0.729 – 1.392) | 0.897 (0.654 – 1.231) | 0.702** (0.508 – 0.967) |
| Receive instrumental support | 1.549 (0.627 – 3.829) | 1.064 (0.790 – 1.433) | 1.143 (0.857 – 1.525) | 1.303* (0.957 – 1.776) |
| Receive emotional support | 0.461* (0.196 – 1.085) | 1.755*** (1.245– 2.472) | 2.379*** (1.704 – 3.320) | 2.078*** (1.463 – 2.952) |

*** p<0.01, ** p<0.05, * p<0.1

Supplementary Table 5. Multinominal logistic regression on Chinese oldest-old’s Life satisfaction with original category (Model 2)

| Variables | Model 2 (OR, 95% CI, Base category = Bad) | | | |
| --- | --- | --- | --- | --- |
|  | Very bad | Average | Good | Very Good |
| Intergenerational support variables | | | | |
| Provide financial support | 0.726 (0.994 – 47.36) | 1.163 (0.836 – 1.619) | 1.349* (0.974 – 1.867) | 1.600*** (1.150 – 2.228) |
| Receive financial support | 0.955 (0.237 – 3.492) | 0.967 (0.685 – 1.364) | 0.884 (0.631 – 1.239) | 0.781 (0.553 – 1.102) |
| Receive instrumental support | 1.536 (0.0370 – 1.109) | 1.375* (0.981 – 1.928) | 1.457** (1.047 – 2.027) | 1.774*** (1.240 – 2.537) |
| Receive emotional support | 0.533 (0.864 – 33.49) | 1.654** (1.122 –2.436) | 2.009*** (1.375 – 2.935) | 1.737*** (1.169 – 2.579) |
| Age | 0.998 (0.853 – 1.020) | 0.983 (0.963 – 1.004) | 1.004 (0.984 – 1.024) | 1.000 (0.979 – 1.021) |
| Gender | 1.123 (0.372 – 6.036) | 0.760* (0.561 – 1.029) | 0.882 (0.656 –1.188) | 0.878 (0.645 – 1.196) |
| Current residence | 1.112 (0.209 – 1.738) | 1.138 (0.874 – 1.483) | 1.023 (0.791 – 1.325) | 1.265* (0.966 – 1.656) |
| Current marital status | | | | |
| Never married, separated and divorced | 4.138 (0.627 – 27.299) | 0.904 (0.436 – 1.875) | 0.739 (0.361 – 1.518) | 0.473* (0.214 – 1.043) |
| Widowed | 2.432 (0.671 – 8.816) | 0.991 (0.684 – 1.437) | 0.910 (0.632 – 1.309) | 0.864 (0.592 – 1.260) |
| Income | 1.000** (1.000 – 1.000) | 1.000 (1.000 – 1.000) | 1.000*** (1.000 – 1.000) | 1.000*** (1.000 – 1.000) |
| Year of schooling | 0.992 (0.804 – 1.224) | 1.030 (0.981 – 1.083) | 1.026 (0.977 – 1.078) | 1.046* (0.995 – 1.099) |
| Number of living children | 1.056 (0.892 – 1.250) | 1.088** (1.014 – 1.169) | 1.117*** (1.042– 1.198) | 1.122*** (1.044 – 1.207) |
| Co–residence | 0.733 (0.228 – 2.363) | 1.150 (0.830 – 1.593) | 1.075 (0.782 – 1.478) | 1.145 (0.825 – 1.591) |
| ADL | 1.220 (0.542 – 2.749) | 0.588*** (0.444 – 0.779) | 0.434*** (0.330 – 0.570) | 0.337*** (0.253 – 0.448) |
| Chronic disease | 0.595 (0.220 – 1.604) | 0.857 (0.571 – 1.286) | 0.727 (0.490 – 1.076) | 0.747 (0.498 – 1.121) |
| Social security insurance | 0.460 (0.160 – 1.325) | 1.093 (0. 658 – 1.813) | 1.118 (0.684 – 1.827) | 1.214 (0.732 – 2.015) |
| Medical insurance | 0.533 (0.194 – 1.460) | 0.858 (0.562 – 1.311) | 0.818 (0.541 – 1.239) | 0.605** (0.396 – 0.925) |
| Old–age insurance | 0.306* (0.086 – 1.087) | 0.898 (0.667 – 1.209) | 0.931 (0.696 – 1.245) | 1.061 (0.785 – 1.434) |
| Community service | 0.924 (0.415 – 2.056) | 1.208 (0.909 – 1.605) | 1.147 (0.869 – 1.513) | 1.188 (0.889 – 1.586) |

*** p<0.01, ** p<0.05, * p<0.1

Supplementary Table 6. Multinominal logistic regression on Chinese oldest-old’s Life satisfaction with original category (Model 4).

| Variables | Model 4 (OR, 95% CI, Base category = Bad) | | | |
| --- | --- | --- | --- | --- |
|  | Very bad | Average | Good | Very Good |
| Intergenerational support variables | | | | |
| Financial support | 0.334 (0.048 – 2.303) | 0.527* (0.250 – 1.110) | 0.400** (0.193 – 0.831) | 0.366** (0.169 – 0.796) |
| Instrumental support | 0.365 (0.034 – 3.915) | 0.539 (0.227 – 1.281) | 0.504 (0.218 – 1.168) | 0.655 (0.268 – 1.600) |
| Emotional support | 0.530 (0.074 – 3.772) | 1.195 (0.560 – 2.548) | 1.309 (0.624 – 2.744) | 1.046 (0.483 – 2.266) |
| Financial and instrumental support | 5.052 (0.632 – 40.390) | 2.980* (0.801 – 11.086) | 2.843 (0.776 – 10.412) | 2.416 (0.636 – 9.176) |
| Financial and emotional support | 0.432 (0.085 – 2.186) | 1.296 (0.665 – 2.525) | 1.382 (0.720 – 2.654) | 1.002 (0.505 – 1.988) |
| Instrumental and emotional support | 0.561 (0.102 – 3.082) | 1.501 (0.735 – 3.064) | 1.576 (0.785 – 3.165) | 1.477 (0.712 – 3.066) |
| Financial, instrumental and emotional support | 0.624 (0.137 – 2.844) | 1.795* (0.918 – 3.509) | 1.953** (1.014 – 3.764) | 1.762 (0.886 – 3.504) |
| Age | 0.995 (0.940 – 1.054) | 0.982* (0.962 – 1.002) | 1.003 (0.984 – 1.023) | 1.000 (0.980 – 1.020) |
| Gender | 1.162 (0.439 – 3.074) | 0.760* (0.555 – 1.041) | 0.885 (0.650 – 1.204) | 0.876 (0.637 – 1.206) |
| Current residence | 1.091 (0.493 – 2.416) | 1.151 (0.875 – 1.515) | 1.049 (0.802 – 1.372) | 1.308* (0.988 – 1.730) |
| Current marital status |  |  |  |  |
| Never married, separated and divorced | 4.308 (0.643 – 28.850) | 0.860 (0.409 – 1.806) | 0.684 (0.328 – 1.427) | 0.429** (0.192 – 0.961) |
| Widowed | 2.338 (0.605 – 9.040) | 0.980 (0.680 – 1.413) | 0.900 (0.629 – 1.289) | 0.859 (0.593 – 1.245) |
| Income | 1.000** (1.000 – 1.000) | 1.000* (1.000 – 1.000) | 1.000*** (1.000 – 1.000) | 1.000*** (1.000 – 1.000) |
| Year of schooling | 0.987 (0.816 – 1.193) | 1.031 (0.979 – 1.086) | 1.029 (0.978 – 1.082) | 1.050* (0.997 – 1.106) |
| Number of living children | 1.043 (0.853 – 1.276) | 1.080** (1.005 – 1.161) | 1.107*** (1.032 – 1.187) | 1.112*** (1.033 – 1.196) |
| Co–residence | 0.705 (0.228 – 2.184) | 1.167 (0.842 – 1.616) | 1.097 (0.798 – 1.508) | 1.176 (0.847 – 1.634) |
| ADL | 1.225 (0.518 – 2.897) | 0.581*** (0.437 – 0.772) | 0.430*** (0.326 – 0.568) | 0.329*** (0.246 – 0.440) |
| Chronic disease | 0.606 (0.224 – 1.641) | 0.879 (0.584 – 1.323) | 0.751 (0.506 – 1.117) | 0.775 (0.515 – 1.168) |
| Social security insurance | 0.434 (0.155 – 1.219) | 1.063 (0.640 – 1.764) | 1.080 (0.660 – 1.769) | 1.175 (0.706 – 1.954) |
| Medical insurance | 0.541 (0.200 – 1.469) | 0.858 (0.560 – 1.315) | 0.811 (0.534 – 1.233) | 0.597** (0.389 – 0.917) |
| Old–age insurance | 0.295* (0.085 – 1.026) | 0.883 (0.655 – 1.189) | 0.916 (0.684 – 1.225) | 1.048 (0.775 – 1.418) |
| Community service | 0.940 (0.426 – 2.074) | 1.209 (0.910 – 1.607) | 1.145 (0.868 – 1.511) | 1.187 (0.889 – 1.585) |

*** p<0.01, ** p<0.05, * p<0.1
